# Beyond Age: Radiological and Inflammatory Factors Associated with Hospital Stay After VATS in Pediatric Complicated Pneumonia—A 10-Year Single-Center Experience

**DOI:** 10.3390/children13101421

**Published:** 2026-10-16

**Authors:** Ihsan Ozdemir, Emre Yasar Karbancioglu, Tugba Akkaya Hocagil, Gulnur Gollu, Ufuk Ates, Ergun Ergun, Ergin Ciftci, Halil Ozdemir, Gul Arga, Tanil Kendirli, Merve Havan, Deniz Tekin

**Affiliations:** 1 Department of Pediatrics, Division of Pediatric Emergency Care, Ankara University School of Medicine, 06620 Ankara, Türkiye; emrekarban@gmail.com (E.Y.K.); tekind@ankara.edu.tr (D.T.); 2 Department of Biostatistics, Ankara University School of Medicine, 06620 Ankara, Türkiye; tahocagil@ankara.edu.tr; 3 Department of Pediatrics, Division of Pediatric Surgery, Ankara University School of Medicine, 06620 Ankara, Türkiye; ggollu@ankara.edu.tr (G.G.); uates@ankara.edu.tr (U.A.); erergun@ankara.edu.tr (E.E.); 4 Department of Pediatrics, Division of Pediatric Infectious Disease, Ankara University School of Medicine, 06620 Ankara, Türkiye; eciftci@ankara.edu.tr (E.C.); ozdemirh@ankara.edu.tr (H.O.); arga@ankara.edu.tr (G.A.); 5 Department of Pediatrics, Division of Pediatric Intensive Care, Ankara University School of Medicine, 06620 Ankara, Türkiye; tkendirli@ankara.edu.tr (T.K.); merve.havan@gmail.com (M.H.)

**Keywords:** video-assisted thoracoscopic surgery (VATS), pediatric empyema, complicated pneumonia, length of hospital stay

## Abstract

**Highlights:**

**What are the main findings?**

- Higher C-reactive protein at presentation was modestly associated with a longer hospital stay after video-assisted thoracoscopic surgery, whereas age was not.
- Most children (82%) were managed with video-assisted thoracoscopic surgery alone, and no conversion to open thoracotomy occurred.

**What is the implication of the main finding?**

- The association of C-reactive protein with length of stay was small and borderline after adjustment for computed tomography, and needs confirmation in larger prospective studies.

**Abstract:**

**Background/Objectives:** We aimed to describe the clinical, laboratory, and radiological characteristics of children with complicated pneumonia/empyema who underwent video-assisted thoracoscopic surgery (VATS) and to identify factors associated with length of hospital stay. **Methods:** We retrospectively reviewed 68 children who presented to the pediatric emergency department of a tertiary university hospital and underwent VATS between April 2015 and April 2025. Factors associated with length of stay were assessed using univariable and multivariable negative binomial regression. **Results:** Median age was 60 months (interquartile range 36–138), and 66% were male. On univariable analysis, C-reactive protein (CRP) was associated with length of stay (incidence rate ratio [IRR] = 1.03 per 100 mg/L; *p* = 0.034), whereas age was not (*p* = 0.542). In the multivariable model, CRP remained borderline associated (IRR = 1.03; 95% confidence interval [CI] 1.00–1.06; *p* = 0.049), whereas performance of computed tomography was not (*p* = 0.142). An atypical (gastrointestinal or pain-predominant) presentation occurred in 25%. *Streptococcus pneumoniae* was the most frequently detected bacterial pathogen, mainly by nasopharyngeal polymerase chain reaction. **Conclusions:** In this single-center, retrospective cohort, higher CRP at presentation, but not age, was modestly associated with a longer hospital stay; this small association needs confirmation in larger prospective studies, and most children (82%) were managed with VATS alone.

## 1. Introduction

Complicated pneumonia refers to pneumonia complicated by parapneumonic effusion, empyema, necrosis, or lung abscess. Empyema progresses through three stages: exudative (stage I), fibrinopurulent (stage II), and organizing (stage III), defined on imaging by the character of the pleural collection and the development of a fibrous pleural peel [1]. *Streptococcus pneumoniae* is the most commonly isolated pathogen in pediatric parapneumonic effusion and empyema, and *Staphylococcus aureus* is an important cause; guidelines recommend blood cultures in hospitalized children and Gram stain and culture of pleural fluid whenever a specimen is obtained [2,3].

Video-assisted thoracoscopic surgery (VATS) is a minimally invasive alternative to thoracotomy, with comparable or better outcomes and fewer musculoskeletal sequelae in children [4,5,6,7]. In complicated pneumonia, VATS allows removal of pleural fluid, debridement, and decortication, and with the uniportal technique the chest tube can be placed through the same incision [8]; it is also used for lung abscess drainage and lung biopsy [6]. Large series report success rates of 91–94%, and complications are mostly minor [9,10].

Pediatric guidelines regard chest-tube drainage with fibrinolytics and VATS as effective options. The guideline of the Infectious Diseases Society of America and the Pediatric Infectious Diseases Society (IDSA/PIDS) recommends VATS when moderate-to-large effusions and respiratory compromise persist after approximately 2–3 days of chest-tube drainage and fibrinolytic therapy [2], whereas the British Thoracic Society (BTS) guideline advises early surgical discussion when drainage, antibiotics, and fibrinolytics fail and notes that there is no evidence on whether early surgery is superior to drainage with fibrinolytics [3]. The optimal timing therefore remains debated [11]; primary thoracoscopic intervention has been associated with shorter chest-tube duration in stage II empyema [12], and hospital stay was longer in more advanced disease stages in a published series [13]. Conversion to open surgery remains a concern, and preoperative pulmonary infection has been associated with a higher risk of conversion [4,14].

The applicability of VATS varies across centers according to equipment, surgical experience, and case volume, and data from tertiary centers in resource-limited settings are limited [15,16]. To our knowledge, the relative contributions of age, inflammatory burden, and radiological extent of disease at presentation to hospital stay after VATS for complicated pneumonia have not been well characterized. We therefore aimed to describe the clinical, laboratory, radiological, and microbiological characteristics of children who presented to the pediatric emergency department of a tertiary university hospital and underwent VATS for complicated pneumonia/empyema between April 2015 and April 2025. We also examined whether age and presenting C-reactive protein (CRP) are associated with length of hospital stay and assessed the frequency of atypical presentations.

## 2. Methods

### 2.1. Study Design

This single-center, retrospective cohort study was conducted at the Department of Pediatric Emergency Medicine, Ankara University Faculty of Medicine, a tertiary referral hospital. Children younger than 18 years who presented between April 2015 and April 2025 and underwent video-assisted thoracoscopic surgery (VATS) for complicated pneumonia/empyema were identified through the hospital information system.

### 2.2. Participants/Study Group

At our center, approximately 250 (±10) children with pneumonia are admitted each year from the pediatric emergency department to the pediatric infectious diseases, pediatric intensive care, or pediatric surgery units. Approximately 87 (±5) of them have bacterial pneumonia. All children in this group who underwent VATS during the study period (*n* = 68) were included. Children managed without VATS were not part of the study cohort.

Complicated pneumonia was defined as pneumonia with parapneumonic effusion or empyema, necrotizing pneumonia, or lung abscess confirmed by imaging. Children were first evaluated in the pediatric emergency department and admitted to the pediatric infectious diseases, pediatric intensive care, or pediatric surgery unit. Treatment began with antibiotics, with or without chest-tube drainage. Pediatric surgery was consulted for children who did not improve clinically or in whom imaging showed loculated or complicated collections, and the decision to perform VATS was made by the pediatric surgery team.

### 2.3. Inclusion Criteria

- Age < 18 years
- Presentation to the pediatric emergency department with pneumonia and subsequent admission to the pediatric infectious diseases, pediatric intensive care, or pediatric surgery unit
- VATS for complicated pneumonia/empyema during the study period

### 2.4. Exclusion Criteria

- VATS performed for an indication other than complicated pneumonia/empyema
- Direct admission by the Department of Pediatric Surgery without presentation to the pediatric emergency department
- Primary open thoracotomy

Complete records were not required for inclusion. No patient was excluded because of missing data; each analysis used the available data for the variables involved.

### 2.5. Data Collection Tools

Data were extracted retrospectively from operative notes, discharge summaries, and laboratory and radiology records in the hospital information system. The following data were recorded:

- Demographic and clinical data: date of birth, sex, age at presentation and at surgery, presenting complaint, initial peripheral oxygen saturation (SpO_2_), respiratory rate and body temperature, duration of fever, physical examination findings, and chronic disease
- Laboratory data at presentation: blood gas (pH, pCO_2_, HCO_3_, lactate), complete blood count (white blood cell, absolute neutrophil and lymphocyte counts, platelets, hemoglobin), and CRP
- Imaging: whether chest radiography, thoracic computed tomography (CT), thoracic magnetic resonance imaging (MRI), and thoracic ultrasound were performed, and the radiological findings
- Microbiology: nasopharyngeal multiplex polymerase chain reaction (PCR) results, blood and pleural fluid culture results, and isolated pathogens
- Treatment and outcome: initial and second admission wards and their durations, length of hospital stay (duration of inpatient treatment), VATS procedure, day of surgery, initial antibiotics, and treatment changes

Laboratory tests were performed on blood samples obtained at the first presentation to the pediatric emergency department, before surgery; only these initial values were analyzed. Serum CRP was measured by an immunoturbidimetric method on a large-scale, fully automated biochemistry autoanalyzer and is reported in mg/L. Venous blood gas parameters (pH, pCO_2_, HCO_3_, lactate) were measured with a blood gas analyzer, and the complete blood count with an automated hematology analyzer, both in the hospital laboratory.

Chest radiography was performed in all 68 patients. Thoracic ultrasound was performed in 23 patients (34%) and thoracic CT in 51 patients (75%); no patient underwent thoracic MRI. Both ultrasound and CT were performed in 12 patients, ultrasound only in 11, CT only in 39, and neither in 6. All imaging was performed before VATS.

Ultrasound was performed in the pediatric emergency department by a pediatric radiologist when pleural effusion was present. CT was performed when clinically indicated, mainly in patients with clinical deterioration, and was reported by a pediatric radiologist. Thoracic CT was performed on a single 16-detector CT scanner in the Pediatric Radiology Division of Ankara University Faculty of Medicine. The main radiological finding was taken from the CT report when CT was performed (*n* = 51) and otherwise from the ultrasound or chest radiography report (*n* = 17). It was categorized as empyema, pleural effusion, consolidation, necrotizing pneumonia/abscess, pneumothorax, or combined/other. In the analyses, imaging variables indicate whether a modality was performed (performed vs. not performed). Imaging reports were prepared as part of routine clinical care by pediatric radiologists; no standardized scoring system or central re-review was applied, and the radiological categories reflect the findings stated in these reports.

Nasopharyngeal samples were obtained at presentation, before antibiotic therapy, and tested by commercial multiplex PCR panels. The viral panel included influenza A (with subtyping) and B, parainfluenza 1–4, coronavirus OC43, NL63, 229E, and HKU1, respiratory syncytial virus A/B, rhinovirus, bocavirus, human metapneumovirus, adenovirus, and enterovirus; the bacterial panel included *Streptococcus pneumoniae*, *Mycoplasma pneumoniae*, *Bordetella pertussis*/*parapertussis*, *Chlamydia pneumoniae*, and *Legionella pneumophila*. Blood cultures were obtained from all patients. Pleural fluid was obtained during VATS and sent for culture; PCR and antigen tests were not performed on pleural fluid. Cultures were processed in the hospital microbiology laboratory using standard methods, and isolates were identified by standard laboratory procedures. Antimicrobial susceptibility results were not analyzed in this study. Microbiological data were collected to describe the cohort and were not analyzed as factors associated with length of stay.

VATS was performed by experienced pediatric surgeons under general anesthesia, with the patient in the lateral position and the affected side up. A 4–5 mm camera trocar was inserted through the intercostal space (4th–6th), often through a previous chest-tube site, and CO_2_ was insufflated at low pressure. Additional trocars were placed as required. Fibrinous adhesions and septations were released under vision, and pleural fluid was sampled. Additional procedures (biopsy, abscess drainage, decortication, pleurodesis, or bullectomy) were performed when required.

Atypical presentation was defined as a primary presenting complaint not referable to the respiratory system, namely gastrointestinal complaints (e.g., abdominal pain, vomiting) or pain-predominant complaints, without cough, tachypnea, or dyspnea as the leading complaint.

Length of hospital stay, the primary outcome, was defined as the duration of inpatient treatment in days until discharge.

### 2.6. Data Analysis

Statistical analyses were performed using R software (R version 4.4.2, R Foundation for Statistical Computing, Vienna, Austria). The distribution of continuous variables was assessed using histograms and the Shapiro–Wilk test. Non-normally distributed variables were summarized as median (first and third quartiles, Q1–Q3), and categorical variables as number and percentage [*n* (%)]. A two-sided *p*-value < 0.05 was considered statistically significant for all tests.

### 2.7. Between-Group Comparisons

Patients were divided into two groups according to the interval between the diagnosis of pneumonia and VATS: early VATS (<7 days) and late VATS (≥7 days). The interval was calculated from the date of diagnosis of pneumonia (first presentation) to the date of VATS. The Wilcoxon rank-sum test was used to compare continuous variables between these groups, and Pearson’s chi-square test or, where expected cell counts were insufficient, Fisher’s exact test was used for categorical variables. For comparisons among the initial wards of admission, the Kruskal–Wallis test was used for continuous variables and Fisher’s exact test for categorical variables.

### 2.8. Length of Hospital Stay Analysis

Length of hospital stay was right-skewed with substantial overdispersion. We therefore assessed factors associated with length of stay using negative binomial regression. Results are reported as incidence rate ratios (IRR) with 95% confidence intervals (CI) and are interpreted as associations; this observational design does not allow causal inference. Variables with *p* < 0.10 in univariable analysis (CRP and whether thoracic CT was performed) were entered into a multivariable model; this selection criterion was defined post hoc and, given the small sample, only two variables were included. Model fit was assessed using the residual deviance and Cook’s distance. Models were fitted with the glm.nb function of the MASS package (code in Appendix A.2).

### 2.9. Recategorization of Variables

Clinical, radiological, and treatment-related variables recorded as free text were regrouped into clinically meaningful categories to improve statistical robustness and clinical interpretability.

## 3. Results

### 3.1. Patient Characteristics

A total of 68 children who underwent VATS for complicated pneumonia were included (Table 1). The median age was 60 months (Q1–Q3: 36–138), and 66% were male. Respiratory symptoms were the predominant presenting complaint (66%). The most common physical examination finding was decreased breath sounds (63%). Seventy-nine percent of the children had no chronic disease.

On laboratory evaluation, the median CRP level was 151 mg/L (Q1–Q3: 85–235). Chest radiography was performed in all patients, thoracic CT in 51 (75%), and ultrasound in 23 (34%). The most common radiological findings were pleural effusion (38%) and necrotizing pneumonia/abscess (34%).

### 3.2. Comparison of Early and Late VATS

Timing was available for 65 of 68 children: 43 had early VATS (<7 days) and 22 had late VATS (≥7 days) (Table 2). The groups did not differ significantly in age, sex, presenting symptoms, physical examination findings, lactate, pH, or white blood cell count. CRP was numerically higher in the late-VATS group (median 174 mg/L vs. 130 mg/L; *p* = 0.066). The main radiological finding differed between the groups (*p* = 0.031). Consolidation was more common in the late-VATS group (27% vs. 2.3%), and pleural effusion was more common in the early-VATS group (49% vs. 23%).

### 3.3. Factors Associated with Length of Hospital Stay

The median length of hospital stay was 16.5 days (Q1–Q3: 14–21). On univariable negative binomial regression (Table 3), age, sex, presence of chronic disease, pH, lactate, and white blood cell count showed no significant association with length of hospital stay; in particular, age was not significantly associated with length of stay (IRR = 0.995 per year; 95% CI 0.979–1.011; *p* = 0.542). CRP level was significantly associated with length of stay (IRR = 1.029 per 100 mg/L; 95% CI 1.001–1.059; *p* = 0.034). The main radiological finding was not significantly associated with length of stay, except for the combined/other category, which was associated with a longer stay than pleural effusion (IRR = 1.38; 95% CI 1.01–1.88; *p* = 0.045); this category included only five patients. Having undergone CT showed a borderline association with longer hospital stay (IRR = 1.19; 95% CI 0.97–1.45; *p* = 0.099).

### 3.4. Multivariable Analysis

After adjustment for whether CT was performed, higher CRP remained borderline associated with longer hospital stay (IRR = 1.026 per 100 mg/L; 95% CI 0.999–1.056; *p* = 0.049), corresponding to an approximately 3% longer stay per 100 mg/L increase. Having undergone CT was not significantly associated with length of stay after adjustment (IRR = 1.16; 95% CI 0.95–1.41; *p* = 0.142) (Table 4).

### 3.5. Ward of Admission and Disease Severity

Children admitted to the intensive care unit were younger (*p* = 0.033), had higher CRP levels (*p* < 0.001), and had a different distribution of radiological findings (*p* < 0.001) than those in the other groups. In the intensive care group, CT was performed in 95% of patients, and necrotizing pneumonia/abscess was the main radiological finding in 68%. The distribution of chronic disease did not differ significantly among the ward-of-admission groups. See Table 5.

### 3.6. Surgical and Treatment Characteristics

The majority of patients (82%) were managed with VATS alone, with additional procedures required in only a limited number of cases (Table 6). The median day of surgery was 4 (Q1–Q3: 2–8). Antibiotic therapy was classified as narrow-spectrum in 69%, broad-spectrum in 10%, and combination therapy (≥3 agents) in 21% of children; a change in antibiotic therapy was required in 56% of patients. No patient required conversion to open thoracotomy.

### 3.7. Use of Imaging Modalities

Imaging was performed at the clinicians’ discretion. Ultrasound was used more often in patients who did not undergo CT (11 of 17, 65%) than in those who did (12 of 51, 24%).

### 3.8. Additional Clinical Findings

Gastrointestinal and pain-predominant symptoms were the presenting complaints in 15% and 10% of the children, respectively. *Streptococcus pneumoniae* was the most frequently detected bacterial pathogen (nasopharyngeal PCR, 24%; pleural fluid, 7.5%). A respiratory virus was detected in 25 of the 67 children with a PCR result (37%), most often influenza A (H1N1) (*n* = 7); 8 of these 25 children also had a bacterial pathogen detected (details in Appendix A.1). The initial antibiotic regimen included ampicillin-sulbactam in 46 patients (68%).

## 4. Discussion

### 4.1. Principal Findings

In this retrospective analysis of 68 children who underwent VATS over ten years, higher CRP at presentation, but not age, was modestly associated with a longer hospital stay. Timing of surgery was associated with the main radiological finding, and children admitted to intensive care had higher CRP and a different distribution of radiological findings.

### 4.2. Patient Characteristics

Our length of hospital stay (median 16.5 days) was considerably longer than that of the 114-patient series by Bishay et al. (7 days) [9]. This difference may reflect differences in case mix, such as the relatively high proportion of necrotizing pneumonia/abscess in our series (34%). Two-thirds of the children were male. Sex was not associated with length of stay or with the timing of surgery in our analyses, and our data do not suggest a specific explanation for this proportion.

### 4.3. Comparison of Early and Late VATS

The higher proportion of consolidation in the late-VATS group (*p* = 0.031) is consistent with disease progression over time. The optimal timing of VATS remains unresolved in the literature [11]. Primary VATS has been associated with a shorter chest-tube duration (3.1 vs. 14.5 days; *p* < 0.05) [12]. Another series similarly found that length of hospital stay increased with advancing disease stage [13]. A multicenter retrospective cohort of patients with empyema also suggested that immediate VATS shortened hospital stay [17].

### 4.4. Factors Associated with Length of Hospital Stay

We found no significant association between age and length of hospital stay (*p* = 0.542). Our data do not show whether disease stage, rather than age, explains the clinical course.

Higher CRP at presentation was associated with a longer hospital stay (*p* = 0.034). The association was small and borderline after adjustment for CT (*p* = 0.049), and should therefore be interpreted cautiously. Among the radiological categories, only the small combined/other category (*n* = 5) was associated with a longer stay than pleural effusion; this isolated finding is based on very few patients and may be due to chance.

### 4.5. Multivariable Analysis

In the multivariable model, the association of CRP with length of stay persisted after adjustment for CT, but it was borderline and the effect was small (about 3% longer stay per 100 mg/L). The association of CT performance with length of stay was attenuated and not significant. Given the sample size and the limited number of variables evaluated, these findings are exploratory and should not be interpreted causally.

### 4.6. Ward of Admission and Disease Severity

Children admitted to intensive care had the highest CRP levels (median 197 mg/L) and the highest proportion of necrotizing pneumonia/abscess (68%), whereas those admitted to the surgical ward had the lowest CRP levels (median 85 mg/L) (Table 5). This gradient is in line with the severity expected at each level of care. As ward allocation reflected clinical judgment, these findings are descriptive.

### 4.7. Surgical and Treatment Characteristics

Most children (82%) were managed with VATS alone, and none required conversion to open thoracotomy. Bishay et al. reported complete resolution in 91% of children [9], although outcome definitions differ between studies. The initial antibiotic regimen included ampicillin-sulbactam in 68% of children, and treatment was changed in 56%. A comparison with other published series is provided in Appendix A.3.

### 4.8. Use of Imaging Modalities

At our center, ultrasound was used mainly in the emergency department when pleural effusion was suspected, whereas CT was reserved for clinical deterioration or when further evaluation was needed. Because imaging was chosen according to clinical need and the findings of the two modalities were not compared, our data do not allow conclusions about their diagnostic agreement. Lung ultrasound has high pooled sensitivity and specificity for pneumonia in children, and single studies suggest accuracy similar to CT for pleural fluid, necrosis, and abscess, but it cannot assess consolidations that do not reach the pleural surface and has not been adopted as a standard imaging method [18]. In a study of 102 hospitalized children with community-acquired pneumonia, lung ultrasound identified pneumonia that was occult on chest radiography in six children (5.9%) [19]. The choice between ultrasound and CT therefore depends on the clinical question and local availability, and should be examined prospectively.

### 4.9. Additional Clinical Findings

Gastrointestinal (15%) and pain-predominant (10%) symptoms were the presenting complaints in a quarter of the children. Complicated pneumonia can therefore present with symptoms that are not referable to the respiratory system.

*Streptococcus pneumoniae* was the most frequently detected bacterial pathogen, and respiratory viruses, particularly influenza A (H1N1), were also common. *S. pneumoniae* was mainly detected by nasopharyngeal PCR, which cannot distinguish colonization from invasive infection, so its etiological role should be interpreted cautiously. Pleural isolation was less frequent (7.5%). Because samples were obtained once at presentation, we cannot determine whether empyema followed a viral infection with bacterial superinfection, or whether viral detection reflected concurrent infection or prolonged shedding. Antimicrobial susceptibility data and data on children who recovered without surgery were not available, so we cannot draw conclusions about antimicrobial resistance or treatment failure.

### 4.10. Clinical Implications

Presenting CRP may be a useful, though small, signal of a longer hospital stay after VATS, but it should be interpreted together with the radiological findings and the clinical course rather than as a stand-alone predictor. Because gastrointestinal or pain-predominant symptoms occurred in a quarter of the children, thoracic disease should be considered in children with such presentations. Because CT and ultrasound were used according to clinical need, our data do not support a preferred imaging strategy.

### 4.11. Strengths

The strengths of this study include the inclusion of all children who underwent VATS for complicated pneumonia at a tertiary center over ten years, the use of explicit definitions for the clinical and radiological variables, laboratory values taken at the first emergency department presentation, and a count-based regression model suited to the skewed distribution of length of stay. The study was also conducted by a multidisciplinary team of pediatric emergency medicine, pediatric surgery, pediatric infectious diseases, pediatric intensive care, and biostatistics specialists.

### 4.12. Limitations

This study has several limitations. First, its single-center, retrospective design limits generalizability and may have led to incomplete records. Second, only children who underwent VATS were included, so we could not compare VATS with non-surgical management or assess why VATS was chosen. Third, the sample size (*n* = 68) limited power to separate the effects of correlated variables such as CRP and radiological findings, so the multivariable model was limited to two variables; one influential observation was identified, and excluding it did not weaken the association between CRP and length of stay (IRR 1.065 per 100 mg/L). Fourth, CT and ultrasound were performed at the clinicians’ discretion, and radiological categories were taken from clinical reports without central review; the exact timing of imaging relative to clinical deterioration was not recorded. Fifth, CRP was a single measurement at presentation. Sixth, practice may have changed over the ten-year period. Finally, the microbiological data came mainly from nasopharyngeal PCR, which cannot separate colonization from infection, and antimicrobial susceptibility data were not available.

### 4.13. Future Research

Larger multicenter, prospective studies are needed to confirm the association between presenting CRP and hospital stay, ideally with standardized radiological reporting, recorded timing of imaging, and comparison with non-surgical management.

## 5. Conclusions

In this single-center, retrospective series of children who underwent VATS for complicated pneumonia/empyema, most were managed with VATS alone and none required conversion to open thoracotomy. Higher CRP at presentation, but not age, was modestly associated with a longer hospital stay; this small, borderline association needs confirmation in larger prospective studies. Gastrointestinal or pain-predominant symptoms occurred in a quarter of the children, so thoracic disease should be considered in children with such presentations.

## Figures and Tables

**Table 1:** Baseline Clinical, Laboratory, and Radiological Characteristics (N = 68).

Characteristic	N = 68
Age (months), median (Q1–Q3)	60 (36–138)
Sex	
Female	23 (34%)
Male	45 (66%)
Initial SpO_2_ (%), median (Q1–Q3)	95.5 (92–96)
Physical examination finding	
Severe respiratory distress	16 (24%)
Other	1 (1.5%)
Normal	3 (4.4%)
Decreased breath sounds	43 (63%)
Rales/Rhonchi	5 (7.4%)
Chronic disease group	
Other	7 (10%)
Cardiac	2 (2.9%)
Neurological	5 (7.4%)
None	54 (79%)
pH (*n* = 38), median (Q1–Q3)	7.37 (7.35–7.41)
Lactate (mmol/L) (*n* = 37), median (Q1–Q3)	1.10 (0.90–1.60)
White blood cell count (/mm^3^), median (Q1–Q3)	12,630 (9570–20,220)
CRP (mg/L), median (Q1–Q3)	151 (85–235)
Imaging performed, *n* (%)	
Chest radiography	68 (100%)
Thoracic CT	51 (75%)
Thoracic ultrasound	23 (34%)
Thoracic MRI	0 (0%)
Main radiological finding, *n* (%)	
Empyema	5 (7.4%)
Combined/Other	5 (7.4%)
Consolidation	7 (10%)
Necrotizing pneumonia/abscess	23 (34%)
Pleural effusion	26 (38%)
Pneumothorax	2 (2.9%)

Values are presented as median (Q1–Q3) or *n* (%). Q1–Q3, first and third quartiles; CT, computed tomography; CRP, C-reactive protein; MRI, magnetic resonance imaging; SpO_2_, peripheral oxygen saturation; WBC, white blood cell. Severe respiratory distress: SpO_2_ < 92% and/or at least one of marked tachypnea, severe chest retractions, nasal flaring, grunting, paradoxical respiratory movements, or intubation at presentation. Other chronic diseases: Down syndrome (*n* = 2), chronic lung disease, post-traumatic splenectomy, PFAPA, febrile convulsion, and VACTERL association with factor VII deficiency and thrombocytopenia. Imaging rows show the number of patients who underwent each examination. The main radiological finding was taken from the CT report when CT was performed (*n* = 51) and otherwise from the ultrasound or chest radiography report (*n* = 17). Main radiological finding categories: Necrotizing pneumonia/abscess: pulmonary parenchymal necrosis (low-attenuation, non-enhancing areas), cavitation, or lung abscess; empyema: pleural collection reported as empyema, without parenchymal necrosis; pleural effusion: pleural fluid, including loculated or septated fluid, without a report of empyema or parenchymal necrosis (consolidation or atelectasis may accompany it); consolidation: parenchymal consolidation without pleural effusion, or with only a small accompanying amount of fluid; pneumothorax: pneumothorax as the main finding; combined/other: several co-equal findings that did not fit the other categories, or other findings. When several findings were present, the most severe was taken as the main finding, in the order necrotizing pneumonia/abscess, empyema, pleural effusion, consolidation. Blood gas analysis was not available at presentation for every patient; pH was available for 38 and lactate for 37 patients (lactate could not be read from one blood gas sample). All other variables were available for all 68 patients.

**Table 2:** Comparison of Early and Late VATS Groups.

Characteristic	Early VATS (<7 Days) N = 43	Late VATS (≥7 Days) N = 22	*p*
Age (months), median	60 (36–132)	78 (15–168)	0.7
Sex			0.091
Female	11 (26%)	11 (50%)	
Male	32 (74%)	11 (50%)	
pH, median	7.37 (7.35–7.41), *n* = 22	7.36 (7.31–7.41), *n* = 14	0.7
Lactate, median	1.10 (0.90–1.90), *n* = 21	0.95 (0.90–1.20), *n* = 14	0.4
White blood cells, median	12,920 (9500–20,740)	11,795 (9640–15,000)	0.2
CRP (mg/L), median	130 (53–206)	174 (143–314)	0.066
Thoracic CT performed	32 (74%)	17 (77%)	>0.9
Thoracic ultrasound performed	16 (37%)	7 (32%)	0.9
Radiological category			0.031
Empyema	3 (7.0%)	2 (9.1%)	
Combined/Other	3 (7.0%)	1 (4.5%)	
Consolidation	1 (2.3%)	6 (27%)	
Necrotizing pneumonia/abscess	13 (30%)	8 (36%)	
Pleural effusion	21 (49%)	5 (23%)	
Pneumothorax	2 (4.7%)	0 (0%)	

Early VATS: VATS performed <7 days after the diagnosis of pneumonia; late VATS: ≥7 days. Timing was available for 65 of 68 patients. Values are median (Q1–Q3) or n (%). Q1–Q3, first and third quartiles (25th and 75th percentiles); n, number of patients. CRP, C-reactive protein; CT, computed tomography; VATS, video-assisted thoracoscopic surgery. Radiological category is the main radiological finding (definitions in the legend of Table 1: necrotizing pneumonia/abscess, empyema, pleural effusion, consolidation, pneumothorax, combined/other). *p* values: Wilcoxon rank-sum test for continuous variables; Pearson’s chi-square or Fisher’s exact test for categorical variables. Blood gas parameters were available only for the number of patients shown in each cell.

**Table 3:** Factors Associated with Length of Hospital Stay (Univariable Negative Binomial Regression).

Variable	IRR	95% CI (Lower)	95% CI (Upper)	*p*
Age (per year)	0.995	0.979	1.011	0.542
Sex (Male)	0.95	0.79	1.14	0.574
Chronic disease: Cardiac	1.24	0.77	2.00	0.376
Chronic disease: Neurological	1.34	0.99	1.82	0.056
Chronic disease: Other	0.83	0.62	1.11	0.215
pH	1.26	0.37	4.28	0.690
Lactate	1.01	0.91	1.13	0.816
White blood cell count (per 1000/mm^3^)	0.997	0.986	1.007	0.538
CRP (per 100 mg/L)	1.029	1.001	1.059	0.034
Thoracic CT performed	1.19	0.97	1.45	0.099
Thoracic ultrasound performed	0.87	0.72	1.04	0.132
Radiological: Combined/Other	1.38	1.01	1.88	0.045
Radiological: Consolidation	1.29	0.98	1.70	0.072
Radiological: Empyema	0.95	0.68	1.33	0.750
Radiological: Necrotizing pneumonia/abscess	1.13	0.93	1.37	0.220
Radiological: Pneumothorax	0.66	0.37	1.15	0.149

IRR, incidence rate ratio for length of hospital stay (per unit increase for continuous variables, or compared with the reference category for categorical variables); CI, confidence interval; CRP, C-reactive protein; CT, computed tomography. Each variable was tested in a separate univariable negative binomial regression model. Units: age, years; CRP, per 100 mg/L; lactate, mmol/L; white blood cell count, per 1000/mm^3^. Thoracic CT and ultrasound: performed vs. not performed. Reference categories: female sex; no chronic disease; radiological category “Pleural effusion”. *n* = 68 for all variables except pH (*n* = 38) and lactate (*n* = 37).

**Table 4:** Factors Associated with Length of Hospital Stay: Comparison of Univariable and Multivariable Negative Binomial Regression.

Variable	Univariable IRR (95% CI)	Univariable *p*	Multivariable IRR (95% CI)	Multivariable *p*
CRP (per 100 mg/L)	1.029 (1.001–1.059)	0.034	1.026 (0.999–1.056)	0.049
Thoracic CT performed	1.19 (0.97–1.45)	0.099	1.16 (0.95–1.41)	0.142

IRR, incidence rate ratio; CI, confidence interval; CRP, C-reactive protein; CT, computed tomography. *n* = 68 for both models; no data were missing for the variables included. The multivariable negative binomial model included CRP (per 100 mg/L) and whether thoracic CT was performed (performed vs. not performed). Analyses were performed in R (MASS::glm.nb).

**Table 5:** Ward of Admission and Disease Severity.

Characteristic	Surgical Ward N = 13	Pediatric Ward N = 33	Intensive Care N = 22	*p*
Age (months), median	96 (72–180)	60 (24–144)	48 (36–96)	0.033
Chronic disease				0.6
Other	2 (15%)	4 (12%)	1 (4.5%)	
Cardiac	0 (0%)	2 (6.1%)	0 (0%)	
Neurological	0 (0%)	2 (6.1%)	3 (14%)	
None	11 (85%)	25 (76%)	18 (82%)	
CRP (mg/L), median	85 (17–112)	152 (87–225)	197 (150–291)	<0.001
Lactate, median	0.80 (0.60–1.90), *n* = 3	1.00 (0.90–1.40), *n* = 15	1.20 (0.90–2.00), *n* = 19	0.3
pH, median	7.40 (7.14–7.46), *n* = 4	7.36 (7.35–7.39), *n* = 15	7.38 (7.32–7.42), *n* = 19	0.8
Thoracic CT performed	5 (38%)	25 (76%)	21 (95%)	0.001
Thoracic ultrasound performed	5 (38%)	13 (39%)	5 (23%)	0.4
Radiological category				<0.001
Empyema	2 (15%)	3 (9.1%)	0 (0%)	
Combined/Other	2 (15%)	2 (6.1%)	1 (4.5%)	
Consolidation	1 (7.7%)	3 (9.1%)	3 (14%)	
Necrotizing pneumonia/abscess	0 (0%)	8 (24%)	15 (68%)	
Pleural effusion	6 (46%)	17 (52%)	3 (14%)	
Pneumothorax	2 (15%)	0 (0%)	0 (0%)	

Ward: first admission ward (Surgical, pediatric surgery ward; Pediatric, pediatric infectious diseases ward; Intensive care, pediatric intensive care unit). Values are median (Q1–Q3) or *n* (%). Q1–Q3, first and third quartiles (25th and 75th percentiles); *n*, number of patients. CRP, C-reactive protein; CT, computed tomography. Radiological category is the main radiological finding (definitions in the legend of Table 1: necrotizing pneumonia/abscess, empyema, pleural effusion, consolidation, pneumothorax, combined/other). *p* values: Kruskal–Wallis test for continuous variables; Fisher’s exact test for categorical variables. Blood gas parameters were available only for the number of patients shown in each cell.

**Table 6:** Surgical and Treatment Characteristics (N = 68).

Characteristic	N = 68
Type of surgical procedure	
VATS alone	56 (82%)
VATS + Biopsy	1 (1.5%)
VATS + Chest tube	5 (7.4%)
VATS + Abscess drainage	1 (1.5%)
VATS + Decortication	2 (2.9%)
VATS + Pleurodesis	2 (2.9%)
VATS + Bullectomy	1 (1.5%)
Time from pneumonia diagnosis to VATS (days), median (Q1–Q3)	4.0 (2.0–8.0)
Length of hospital stay (days), median (Q1–Q3)	16.5 (14.0–21.0)
Antibiotic spectrum	
Narrow spectrum	47 (69%)
Broad spectrum	7 (10%)
Combination (≥3 agents)	14 (21%)
Change in treatment	
No	30 (44%)
Yes	38 (56%)

VATS, video-assisted thoracoscopic surgery; VATS + procedure indicates an additional procedure performed during the same operation. Time from pneumonia diagnosis to VATS is given in days. Antibiotic spectrum was classified according to the pediatric infectious diseases department protocols (narrow-spectrum, broad-spectrum, or combination therapy with ≥3 agents). Change in treatment: change of the initial antibiotic regimen during follow-up. Values are median (Q1–Q3) or *n* (%). Q1–Q3, first and third quartiles (25th and 75th percentiles); *n*, number of patients.

## Data Availability

The data presented in this study are available on request from the corresponding author. The data are not publicly available due to privacy and ethical restrictions related to pediatric patient information.

## Appendix A

### Appendix A.1. Supplementary Table: Microbiological and Clinical Details

The detailed distribution of the microbiological profile summarized in the Results section of the main text is presented below.

**Respiratory Virus (Nasopharyngeal PCR, *n* = 67)**	***n* (%)**
Not detected	42 (63%)
Influenza A (H1N1)	7 (10%)
Rhinovirus	3 (4.5%)
Bocavirus	4 (6.0%)
Metapneumovirus A/B	3 (4.5%)
RSV A/B	2 (3.0%)
Parainfluenza 1–4	4 (6.0%)
Coronavirus (OC43)	1 (1.5%)
Enterovirus	1 (1.5%)
Respiratory viruses detected by multiplex polymerase chain reaction (PCR) in nasopharyngeal samples obtained at presentation. One patient had no result (*n* = 67). Values are *n* (%). RSV, respiratory syncytial virus.	

**Distribution of Bacterial/Other Pathogens (Nasopharyngeal PCR, Pleural Fluid and Blood Cultures, *n* = 67)**

**Pathogen**	**n (%)**
Not detected	42 (63%)
*Streptococcus pneumoniae* (nasopharyngeal PCR)	16 (24%)
*Streptococcus pneumoniae* (pleural)	5 (7.5%)
*Streptococcus* anginosus (pleural)	1 (1.5%)
*Legionella pneumophila*	1 (1.5%)
Blood culture: *Pseudomonas*	1 (1.5%)
Blood culture: Acinetobacter baumannii	1 (1.5%)
Bacterial pathogens detected by multiplex PCR in nasopharyngeal samples, by pleural fluid culture (obtained during VATS), and by blood culture. One patient had no result (*n* = 67). Values are *n* (%). PCR, polymerase chain reaction; VATS, video-assisted thoracoscopic surgery.	

Rare/isolated pathogens (*n* = 67): *Staphylococcus aureus*, Burkholderia cepacia, coagulase-negative staphylococcus, *Moraxella catarrhalis*, Stenotrophomonas maltophilia, *S. pyogenes* (pleural), and metapneumovirus/parainfluenza co-infection, each observed in a single case (1.5%).

Of the 8 children with viral and bacterial co-detection, *S. pneumoniae* was detected by nasopharyngeal PCR in 4 and in pleural fluid in 3. Of the 7 children with influenza A, 3 had *S. pneumoniae* detected by nasopharyngeal PCR.

### Appendix A.2. Statistical Analysis Code

The R code for the multivariable negative binomial regression model is presented below for reproducibility.

library(MASS); data$CRP100 <- data$CRP/100 # CRP per 100 mg/L

model_multi <- glm.nb(LOS ~ CRP100 + CT, data = data) # LOS: length of hospital stay (days)

summary(model_multi); cooks.distance(model_multi)

exp(coef(model_multi)); exp(confint(model_multi))

### Appendix A.3. Comparison of Our Study with Comparable Series in the Literature

**Study**	**Country**	***n***	**Indication**	**Success/Cure Rate**	**Conversion**	**Hospital Stay (Days)**
Our study	Turkey	68	Complicated pneumonia/empyema	—	—	16.5 (median; 14–21)
Bishay et al. [9]	United Kingdom	114	Empyema	91% complete resolution	7%	7 (median)
Ateş et al. [10]	Turkey	34	Empyema (stage 2–3)	94% cure	—	32.5 (median, total)
Rodriguez et al. [12]	USA	13	Empyema (stage II)	—	—	3.1 (primary)/14.5 (delayed)
Thori et al. [13]	India	33	Empyema (stage I–III)	—	3.03%	4/5.1/9.3 (by stage)
Shivachev et al. [16]	Bulgaria	87 (41 VATS)	Parapneumonic complication	—	—	—
“—”: data not reported or not presented in a comparable format in the respective study. *n*, number of patients; VATS, video-assisted thoracoscopic surgery; median values are followed by the first–third quartile range where reported.						
